# Advances in Teaching Phacoemulsification: Technologies, Challenges and Proposals

**DOI:** 10.5935/0004-2749.2023-1005

**Published:** 2023

**Authors:** Newton Kara Junior

**Affiliations:** 1 Universidade de São Paulo, São Paulo, SP, Brazil

Democratization of medical knowledge, new information technologies and simulated surgical training have revolutionized the teaching of phacoemulsification. However, it has never been so difficult to learn to operate during medical residency.

Until the 1980s, in general, for physicians to keep updated with the main advances in Medicine, it was necessary to travel abroad. The reality that prevailed in national scientific meetings was that of complex presentations, which did not make other doctors able to perform better. Even in teaching hospitals, the mentality was often to teach everyone, but not pass on all the information. Medical knowledge was restricted and those who had it, in general, tried to show that they had it, but did not fully transmit it. There was a perception that this knowledge was the main professional differential^(1)^.

Currently, in academic circles, there is practically no reservation of knowledge anymore. With the advent of the internet, information and updates are easily available to teachers and students alike. In this way, the teacher is often faced with students who have already studied the subject of the class and may occasionally even know more about the issue than the outdated professor. Active teaching methodologies, such as problem-based learning (PBL), in which the student constructs knowledge from online sources with subsequent group discussion, assisted by facilitators, although still little used in specialization, not only offer better learning conditions than those of traditional lectures, but also compensate asymmetries in the academic resources of several educational institutions. Even so, if the student realizes that he cannot access information in his teaching hospital, it is possible to acquire knowledge in courses, meetings or even online.

Another important resource for learning phacoemul-sification is simulation-based training, which is becoming popular for educational purposes among residents in Ophthalmology. The purpose of this type of training is to guarantee safety and proper training before transferring skills to real patients^(2)^.

There are many artificial eyes and computerized simulators for surgical training, but the main barrier is cost. A novelty that will make Dry-labs promising are artificial eyes with national technology, an important innovation that will allow, by reducing the cost of training, to spread this teaching tool and facilitate the learning of phacoemulsification. OrbiTau® is a low-cost artificial eye, developed together with the University of São Paulo (USP-SP), which enables simulated training of the main stages of phacoemulsification, under realistic conditions and with the prospect of being made feasible for specialization courses.

However, even with so many educational resources, we found that most residents in Brazil finish specialization without being sufficiently prepared to perform phacoemulsification and those who seek the skill need to do fellowships to supplement their training.

One of the reasons is lack of surgeries for training residents, resulting from interruption of the National Cataract Campaign (CNC), where the government financed, unrestrictedly, cataract surgeries performed in teaching hospitals. In 2006, the Ministry of Health withdrawn the CNC, thus, teaching hospitals had limited quotas for performing surgeries. At USP-SP, for example, where 5,078 surgeries were performed in 2005, due to imposed budget limitation, the number of surgeries performed in 2015 was only 3,902 procedures. A similar situation was observed in other teaching hospitals^(3)^.

Since 2009, the main government health project for the treatment of cataract was through bidding, in which private companies negotiated the lowest value to carry out quotas for thousands of surgeries. Changing the model of public funding for cataract surgeries, with the replacement of teaching hospitals by the private sector, caused damage to the training of new surgeons^(4)^.

Another issue to be discussed is whether three years of specialization are enough to learn how to safely and accurately operate cataract of different degrees of difficulty. It depends. If learning is optimized with adequate theoretical and surgical guidance and the teaching system is structured and planned, yes. Otherwise, the student himself will not feel safe to operate difficult cases or with no supervision. He will be looking for at least two more years of fellowship to fill the gap.

The ideal time to train surgery is during specialization, where there is no lack of teachers, patients, equipment and the student’s only priority is to learn. As the COVID-19 pandemic and new government strategies for granting quotas for cataract surgery have reduced the number of surgeries performed at teaching hospitals, where most of the training for new surgeons takes place, residents to learn how to do phacoemulsification need to get organized and optimize their learning.

Our proposal is that the teaching of phacoemulsification should start from the beginning of the first year of specialization and consist of:

1. At least 40 hours of theory on the surgical techniques and technology used in surgery, with active teaching methodologies;
2. Reading textbooks;
3. At least 20 hours of video discussion where there is instructive surgical situations and resolution of complications;
4. At least 10 hours of training on simulators and artificial eyes; and
5. Active participation in assisted surgeries, with attention to the functioning of the phacoemulsifier and the impact of its fluidic functions on the surgery.

Once this instructive sequence has been completed, the final stage, which is the effective performance of surgery under the supervision of the guiding surgeon, will have the specific purpose of training manual skills and putting into practice the knowledge already acquired.

In this way, the teaching of phacoemulsification would be optimized, and the student-surgeon would be able to evolve consistently after each procedure, as he would be able to recognize the reason for his difficulties, analyze the solution suggested or performed by the supervisor and think about his actions in following surgeries. Surgeons who do not realize that they have had difficulties at some stage or who are unable to identify the reason for their complications will find it difficult to learn from their own errors and successes, and will need to do many more surgeries to learn the technique.

Having in mind this above system, the student-surgeon would be able to effectively evolve after each procedure and would optimize his learning, saving his time and valuing the institution’s resources. With this model, we have realized that performing about 70 surgeries, in general, is enough to achieve surgical self-sufficiency. Any additional surgeries would aim to refine the technique, improve results and make difficult cases less difficult^(5)^.

Thus, we reinforce that if the resident does not make an effort from the beginning of specialization and only in the last year of the residency appears in the surgical center to start learning, he will probably have to do about 200 surgeries to reach self-sufficiency. If the student does not have that “surgical volume” available, he will need to do a fellowship to improve himself, which will make the specialized training more expensive and time-consuming, or this surgical activity will have to be abandoned.

No matter how good the professor is, in the surgical center, when supervising the surgery, in cases where the student-surgeon does not yet know the technique, the technology and has not previously trained in some surgical maneuvers, the teacher will only be a “firefighter” to resolve complications.

We consider that the effectiveness of the surgical teaching model based more on practice than on theory raises questions and deserves further debate.

As currently there is no more reserve of knowledge and the structural conditions to perform surgeries are not difficult to access, the variable that will determine the success of learning phacoemulsification will be the time available to dedicate to the project. The perception of having or not having time is “tricky”, because during the specialization period, time is abundant and “cheap”, but as soon as the residency ends, it becomes scarce and “expensive”. Thus, to optimize learning during residency, it is essential to be able to manage time and choose the most effective ways of acquiring knowledge.

In surgical learning, the attitude of students is important, because while teaching is the responsibility of educators, learning is a competence of the student. We consider that there would be no need for fellowships in cataract surgery if teaching institutions were better prepared and students made more effort to teach/learn phacoemulsification during residency.
